# Targeting PI3Kβ alone and in combination with chemotherapy or immunotherapy in tumors with PTEN loss

**DOI:** 10.18632/oncotarget.27503

**Published:** 2020-03-17

**Authors:** Nicci Owusu-Brackett, Ming Zhao, Argun Akcakanat, Kurt W. Evans, Erkan Yuca, Ecaterina Ileana Dumbrava, Filip Janku, Funda Meric-Bernstam

**Affiliations:** ^1^ Department of Surgical Oncology, The University of Texas MD Anderson Cancer Center, Houston, TX 77030, USA; ^2^ Department of Investigational Cancer Therapeutics, The University of Texas MD Anderson Cancer Center, Houston, TX 77030, USA; ^3^ Department of Breast Surgical Oncology, The University of Texas, MD Anderson Cancer Center, Houston, TX 77030, USA; ^4^ The Sheikh Khalifa Bin Zayed Al Nahyan Institute for Personalized Cancer Therapy, The University of Texas MD Anderson Cancer Center, Houston, TX 77030, USA

**Keywords:** PI3Kβ, PTEN loss, AZD8186, chemotherapy, immunotherapy

## Abstract

**Background:** PTEN-deficient tumors are dependent on PI3Kβ activity, making PI3Kβ a compelling target. We evaluated the efficacy of PI3Kβ inhibitor AZD8186 on tumors with PTEN loss.

**Results:** *In vitro* cell viability assay and immunoblotting demonstrated that PTEN loss was significantly correlated with AZD8186 sensitivity in triple negative breast cancer (TNBC) cell lines. Colony formation assay confirmed sensitivity of PTEN-deficient cell lines to AZD8186. AZD8186 inhibited PI3K signaling in PTEN loss TNBC cells. AZD8186 in combination with paclitaxel, eribulin had synergistic effects on growth inhibition in PTEN loss cells. AZD8186 promoted apoptosis in PTEN loss cells which was synergized by paclitaxel. *In vivo*, AZD8186 had limited activity as a single agent, but enhanced antitumor activity when combined with paclitaxel in MDA-MB-436 and MDA-MB-468 cell-line xenografts. AZD8186 significantly enhanced antitumor efficacy of anti-PD1 antibodies in the PTEN-deficient BP murine melanoma xenograft model, but not in the PTEN-wild-type CT26 xenograft model.

**Methods:** *In vitro*, cell proliferation and colony formation assays were performed to determine cell sensitivity to AZD8186. Immunoblotting was performed to assess PTEN expression and PI3K signaling activity. FACS was performed to evaluate apoptosis. *In vivo*, antitumor efficacy of AZD8186 and its combinations were evaluated.

**Conclusions:** AZD8186 has single agent efficacy in PTEN-deficient TNBC cell lines *in vitro*, but has limited single agent efficacy *in vivo*. However, AZD8186 has enhanced efficacy when combined with paclitaxel and anti-PD1 *in vivo*. Further study is needed to determine optimal combination therapies for PTEN-deficient solid tumors.

## INTRODUCTION

Phosphatidylinositol 3-kinase (PI3K)/AKT/mTOR pathway is an important regulator of many physiological cellular processes that promote differentiation, proliferation and survival of a normal cell [1]. In cancer, deregulation of this pathway results in increased cell proliferation, survival, motility, dysregulated metabolism and decreased autophagy contributing to the pathogenesis of cancer [2, 3]. Phosphatase and tensin homolog (PTEN) is a negative regulator of the PI3K pathway, and maintains balanced cell differentiation, proliferation and survival [4]. Mutations, loss of copy number, epigenetic silencing and downregulation of PTEN protein by miRNA can result in PTEN function inactivation, leading to activation of PI3K/AKT/mTOR pathway, which subsequently increases tumor growth, invasion and metastasis across a diverse set of solid tumors including breast, endometrial, prostate, renal cell, hepatocellular, glioblastoma and colorectal cancers [5, 6]. Loss of PTEN and increased PI3K signaling are associated with resistance to trastuzumab and endocrine therapy in hormone receptor positive breast cancer and with poor prognosis in triple negative breast cancers (TNBC) [7–10].

PI3K family is composed of four classes (I, II, III, and IV) of intracellular signal transducer enzymes capable of phosphorylating phosphatidylinositol. PI3K-p110α, PI3K-p110β, PI3K-p110γ, and PI3K-p110δ (PI3Kα, PI3Kβ, PI3Kγ, and PI3Kδ) are the members of class I. Recently, Wee *et al* demonstrated that PTEN-deficient tumors are dependent on PI3Kβ catalytic isoform activity [11]. *In vitro*, they revealed significant growth inhibition of PTEN-deficient tumors by depleting *PIK3CB* which encodes PI3Kβ, while no such growth inhibition effect was shown in corresponding PTEN-deficient tumors with downregulation of *PIK3CA* or *PIK3CD* encoding PI3Kα and PI3Kδ, respectively. The pathway inactivation and subsequent growth inhibition as a result of downregulation of PI3Kβ isoform were confirmed *in vivo*. Thus, PI3Kβ isoform is the driver of abnormal proliferation in PTEN-null cancers, and as such, PI3Kβ is a promising target for therapy in PTEN-deficient TNBC.

AZD8186 is a selective and potent small-molecule inhibitor of PI3Kβ, with additional activity against PI3Kδ isoform [12–14]. Its antitumor activity has been demonstrated in PTEN-deficient prostate, squamous lung carcinoma and germinal-center diffuse large cell B-cell lymphoma preclinical models [12, 15]. This raises the possibility that AZD8186 may have a role in TNBC with PTEN loss as well.

Mittendorf *et al*. reported that PTEN loss increased PD-L1 expression in TNBC [16]. In melanoma patients, PTEN loss was associated with resistance to immune checkpoint inhibitors [17]. Further, the combination with PI3Kβ inhibitor GSK2636771, but not with pan-PI3K inhibitor BKM120, was found to enhance the antitumor activity of immune checkpoint inhibitors [17].

We sought to determine the antitumor efficacy of AZD8186 as a single agent and in combination with standard chemotherapeutic agents in TNBC cell lines with varying PTEN status. *In vivo* confirmation of *in vitro* identified combinations was performed. Further, the antitumor efficacy of AZD8186 in combination with anti-PD1 inhibitor was evaluated in syngeneic mouse models of varying PTEN status and tumor types.

## RESULTS

### AZD8186 has antitumor efficacy in TNBC cells with PTEN loss *in vitro*

**Figure 1:**
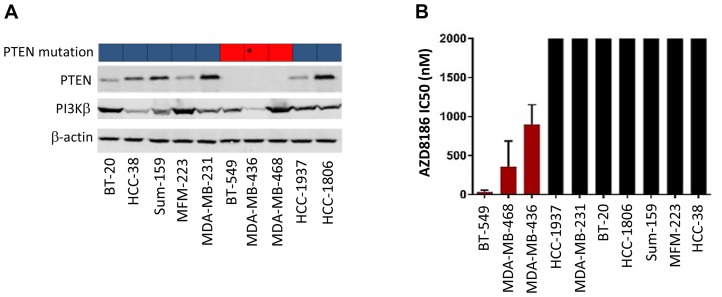
Effects of AZD8186 on cell proliferation *in vitro*. (**A**) PTEN mutation and protein expression status. Top blocks: PTEN mutation status data from COSMIC. Blue: wild-type; red: mutation. ^*^Data from research publication [18]: MDA-MB-436 cells have a gross PTEN mutation (intragenic copy number increases) causing PTEN protein loss. Cells were lysed and immunoblotted after SDS-PAGE for PTEN and PI3Kβ with indicated antibodies. (**B**) Cell proliferation assay. Ten TNBC cell lines were treated with AZD8186 at 6 concentrations of a five-fold dilution series for 72 hours. Cell viability was measured using the sulforhodamine B assay, and half-maximal inhibitory concentration (IC_50_) was then calculated using isobologram curves. Sensitivity was defined as IC_50c<c_1000 nM (mean ± SD). The CI_50s_ that are over 2000 nM were shown at cutoff 2000 nM in the figure.

To evaluate the antitumor efficacy of AZD8186 in TNBC, we tested AZD8186 sensitivity in a panel of 10 TNBC cell lines with varying PTEN genotypes. First, we examined expression of PI3Kβ, the target of AZD8186, in these cell lines. Immunoblotting showed that all these TNBC cell lines, including the PTEN null cell lines, expressed PI3Kβ at various levels (Figure 1A). Western blot showed that PTEN expression is lost in three cell lines, BT-549, MDA-MB-468, and MDA-MB-436, while other seven cell lines expressed PTEN at various levels (Figure 1A). The PTEN status in these cell lines are consistent with previous reports and the database of Catalogue of Somatic Mutations in Cancer (COSMIC). The COSMIC database showed that among these TNBC cell lines, BT-549 and MDA-MB-468 cells have deletion-frameshift mutations (Figure 1A). Previous studies have reported that the regions of apparent PTEN loss of heterozygosity in MDA-MB-436 cells contained highly focal, intragenic copy number increases (CNIs) that affect a portion of intron 2. This is associated with loss of PTEN protein on western blotting [18]. Of note, HCC-1937 cell line was reported to have PTEN mutation [18, 19]. It was the only cell line with a *PTEN* mutation that did not result in loss of PTEN protein.

Next, we determined the sensitivity of these TNBC cell lines to AZD8186 on cell survival. It has been previously reported that in patients receiving AZD8186 with the recommended dose of 60 mg BID 5 days on, 2 days off, the maximum plasma concentration obtained was 500 ng/ml, which is equivalent to 1.09 μM [20]. Given this clinically achievable dose and previous *in vitro* studies [12], we defined that the cell lines with IC_50_ less than 1 μM are sensitive to AZD8186. Cell lines were treated for 72 hours with DMSO or AZD8186. Cell growth was measured using SRB colorimetric assay. IC_50_ calculated by dose-response isobologram curves was used to evaluate sensitivity. The results showed that out of ten TNBC cell lines, three (BT-549, MDA-MB-468, and MDA-MB-436) were sensitive to AZD8186 with IC_50_ of 31, 358, and 899 nM, respectively (Figure 1B, Supplementary Table 1). Other seven cell lines were not responding to AZD8186 treatment as their IC_50s_ were all over 2 μM (Figure 1B, Supplementary Table 1).

**Figure 2:**
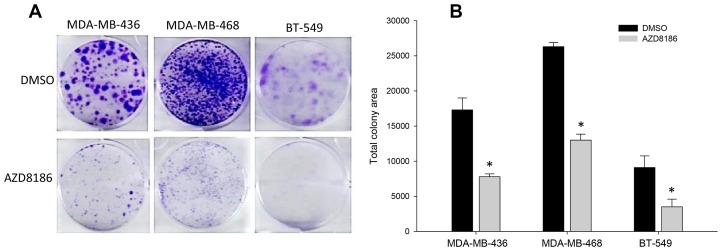
Effect of AZD8186 on colony formation ability. (**A**) Colony formation assay. TNBC MDA-MB-436, MDA-MB-468, and BT-549 cells were seeded in 6-well plates in triplicate for each treatment group. Cells were treated with DMSO or AZD8186 at 1 μM for 2 weeks, with refreshing drug every 3-4 days. Crystal violet-stained colonies were imaged and scanned. (**B**) Colony quantitation. Total colony area was quantitated using Image J v.1.48 software. Values presented as mean ± SD are obtained from triplicate wells from single experiment. ^*^ *p* values for AZD8186 vs DMSO control (*p* < 0.01 for all three cell lines).

Using colony formation assays, we confirmed the sensitivity of the three PTEN-deficient cell lines, MDA-MB-436, MDA-MB-468, and BT-549, to AZD8186. Cells were treated with DMSO or AZD8186 at 1 μM every other 3-4 days for two weeks. Colony staining showed that AZD8186 treatment substantially reduced the capability of all these cell lines to form colonies, compared to the vehicle controls (Figure 2A). Quantitation results demonstrated that cells with AZD8186 treatment had significantly less total colony area than control in all the three cell lines (*p <* 0.01) (Figure 2B). Together with the results of cell proliferation assay above, these findings suggest that AZD8186 is capable of inhibiting cell growth of TNBC cells that are deficient in PTEN.

### AZD8186 inhibits Akt signaling

**Figure 3:**
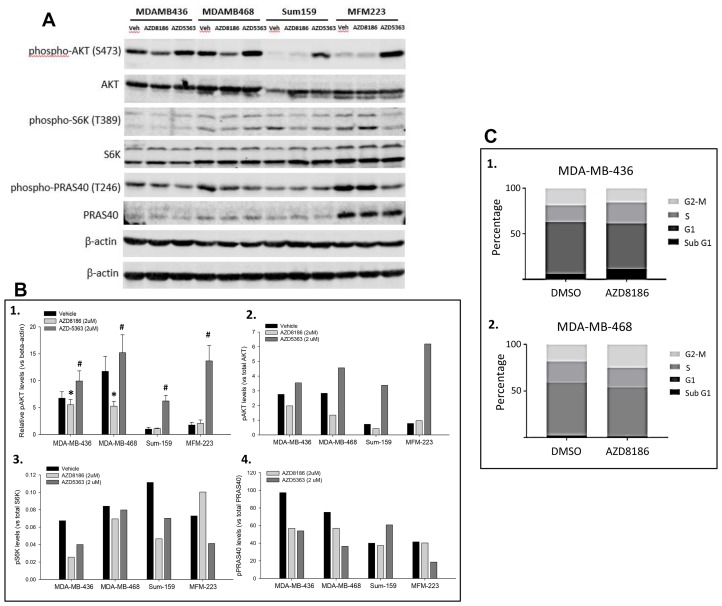
Effects of AZD8186 on cell signaling and cell cycle. (**A**) Immunoblotting of PI3K pathway. PTEN-loss MDA-MB-436 and MDA-MB-468 cells and PTEN-wild-type Sum-159 and MFM-223 cells were treated with DMSO or AZD8186 at 2 μM for 2 hours. Cell lysates were loaded for SDS-PAGE and blotted with the indicated antibodies. Top β-actin panel is a loading control for phosphoproteins, and bottom β-actin panel is a loading control for non-phosphoproteins. (**B**) Quantitation of immunoblotting. Relative phospho-AKT levels were quantitated by normalizing with β-actin (B-1). Mean ± SD values were obtained from 4 independent experiments. ^*^AZD8186 vs vehicle, MDA-MB-436: *p =* 0.037; MDA-MB-468: *p =* 0.0002; #: AZD5363 vs vehicle, *p =* 0.003, 0.036, 0.000001, and 0.000003 for MDA-MB-436, MDA-MB-468, Sum-159, and MFM-223, respectively. Phosphorylated proteins were also normalized by total proteins of AKT (B-2), S6K (B-3), and PRAS40 (B-4), respectively. (**C**) FACS analysis of cell cycle. MDA-MB-436 cells (C1) and MDA-MB-468 cells (C2) were treated with DMSO or AZD8186 at 1 μM for 72 hours. Cell cycle phases were determined with propidium iodide by fluorescence-activated cell sorting.

To evaluate the mechanism of action of AZD8186 in PTEN-deficient tumors, we assessed its effect on PI3K signaling. Four TNBC cell lines, including PTEN loss MDA-MB-436, MDA-MB-468 cell lines and non-PTEN loss Sum-159, MFM-223 cell lines, were evaluated. The TNBC cells were treated with AZD8186 at 2 μM, or DMSO for 2 hours. Immunoblotting showed that compared to the vehicle control, treatment with AZD8186 moderately but significantly decreased levels of phospho-AKT in both PTEN loss cell lines, normalized by β-actin (*p =* 0.037 and 0.0002, respectively) (Figure 3A, 3B-1). On the other hand, in both non-PTEN loss cell lines, AZD8186 did not reduce phospho-AKT levels (Figure 3A, 3B-1). These phospho-AKT changes were also seen by normalization with total AKT levels (Figure 3B-2). Normalization with total proteins also showed that AZD8186 inhibited phosphorylation of S6K and PRAS40 which are downstream targets of AKT in the PTEN loss cell lines (Figure 3A, 3B-3, 3B-4). In non-PTEN loss cell lines, except phospho-S6K in Sum-159 cells, AZD8186 did not exhibit an inhibitory effect on phosphorylation of S6K and PRAS40 (Figure 3A, 3B-3, 3B-4). In order to validate drug efficacy and immunoblotting, we compared AZD8186 with AKT inhibitor AZD5363. We found that AZD5363 enhanced phospho-AKT levels in all the four cell lines, particularly striking in non-PTEN loss cells (Figure 3A, 3B-1, 3B-2), but AZD5363 decreased phospho-S6K and phospho-PRAS40 levels in some of these cell lines (Figure 3A, 3B-3, 3B-4).

The effect of AZD8186 on cell-cycle progression was evaluated in MDA-MB-436 and MDA-MB-468 cell lines. The cells were treated with AZD8186 at 1 μM or DMSO for 48 hours and subsequently harvested. Percentage of cells in G1, S and G2-M phases of the cell cycle were determined by flow cytometry using propidium iodide. We found that AZD8186 did not induce significant changes in the percentage of each of cell cycle phases (Figure 3C-1, 3C-2).

### AZD8186 in combination with paclitaxel has enhanced antitumor efficacy *in vitro*

**Figure 4:**
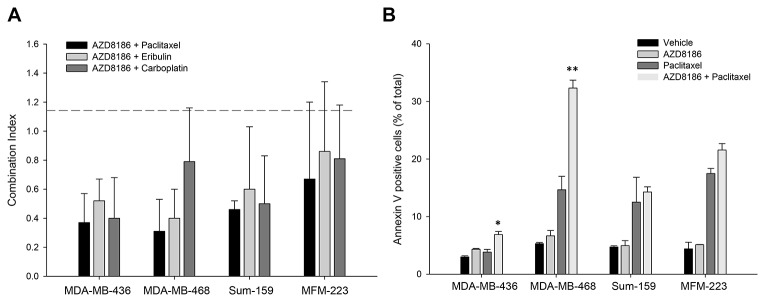
Effects of AZD8186 in combination with standard chemotherapy *in vitro*. (**A**) Cell viability assay. The TNBC cell lines were treated with AZD8186 in combination with paclitaxel, eribulin, and carboplatin for 72 hours. Sulforhodamine B staining was performed to measure cell viability rate. Combination index (CI) was then calculated using the method of Chou and Talalay. CI values: <0.8: synergism; 0.8 – 1.2: addition; and >1.2: antagonism. Most mean ± SD values were obtained from 4 experiments. (**B**) Cell apoptosis assay. Cells were incubated with AZD8186, paclitaxel and their combination for 72 hours. The floating and attached cells were collected and stained with annexin V, followed by flow cytometry analysis. Percentage of annexin V-positive apoptotic cells in total cells were presented (mean ± SD). ^*^ *p =* 0.029 (combination vs paclitaxel alone); ^**^ *p =* 0.006 (combination vs paclitaxel alone).

To determine the effects of AZD8186 in combination with standard chemotherapy, we selected three chemotherapeutic agents commonly used for breast cancer therapy: paclitaxel (microtubule stabilizer), carboplatin (DNA-binding alkylating agent) and eribulin (microtubule inhibitor). MDA-MB-436, MDA-MB-468, Sum-159, and MFM-223 cells were treated with serial concentration dilutions of AZD8186 in combination with serial dilutions of the three various chemotherapy agents. After 72 hours of treatment, growth inhibition was assessed with SRB assay, and IC_50_ was calculated for single agent treatment alone and the combination. Combination index (CI) values were then calculated using the Chou-Talalay method, where a CI value <0.8 indicates synergism, 0.8 to 1.2 indicates addition and a CI greater than 1.2 indicates antagonism [21, 22]. The CI values showed that most of the combinations of AZD8186 with paclitaxel, eribulin, or carboplatin produced marked synergistic effects on inhibition of cell proliferation in MDA-MB-436, MDA-MB-468, and Sum-159 cells, with CI values around and below 0.5 (Figure 4A, Supplementary Table 2). Compared to these three cell lines, the therapeutic efficacy of these combinations were reduced in non-PTEN loss MFM-223 cells, where the synergisms were moderate with CIs around 0.8 (Figure 4A, Supplementary Table 2).

Next, we examined AZD8186 combination efficacy on apoptosis. Apoptosis status was evaluated by quantitation of annexin V-positive cells using fluorescence-activated cell sorting (FACS). The above four cell lines were treated with AZD8186 at 2 μM, or paclitaxel at 5 nM, or both for 3 days. The results showed that AZD8186 moderately induced annexin V-positive apoptotic cells in both PTEN-loss MDA-MB-436 and MDA-MB-468 cells, but not in non-PTEN-loss cells (Figure 4B). However, when paclitaxel was combined with AZD8186, it significantly enhanced AZD8186 efficacy in both PTEN-loss cell lines, particularly in MDA-MB-468 cells where the combo treatment induced 33% apoptotic cells compared to about 10% apoptotic cells in single drug treatments, *p* = 0.006 (Figure 4B). We did not observe enhanced apoptosis in non-PTEN-loss cell lines.

### AZD8186 in combination with paclitaxel has enhanced antitumor efficacy *in vivo*

**Figure 5:**
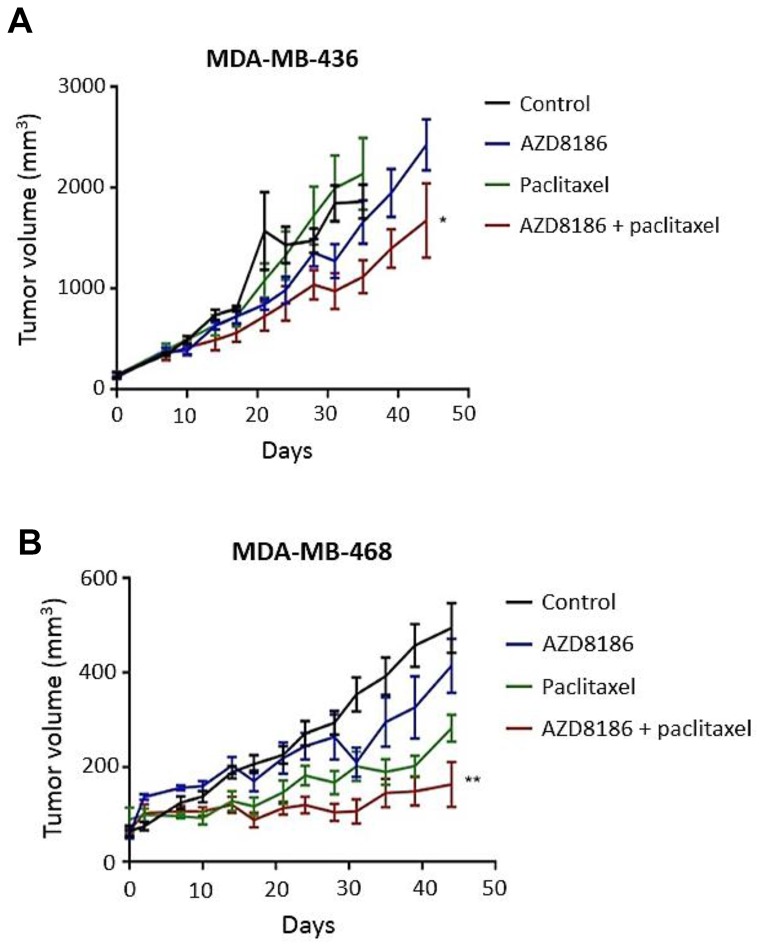
Effects of AZD8186 in combination with paclitaxel on tumor growth of TNBC cell line-derived xenograft models. MDA-MB-436 cells (**A**) and MDA-MB-468 cells (**B**) were subcutaneously inoculated into nude mice. When tumors reach at least 100 mm^3^, mice were treated with vehicle, AZD8186 at 50 mg/kg daily, paclitaxel at 10 mg/kg, once a week or AZD818 in combination with paclitaxel. Tumor volume was measured at the indicated time points. Mean ± SEM values of tumor volume were obtained from 5 mice in each treatment group from single experiment. The tumor volumes at the conclusion of experiment were analyzed by two-way analysis of variance to determine statistical significance. ^*^ *p* values for MDA-MB-436 cell model (combination vs. control: *p =* 0.0049; combination vs Paclitaxel: *p <* 0.001; combination vs AZD8186: *p =* 0.0104). ^**^ *p* values for MDA-MB-468 cell model. (combination vs control: *p <* 0.001; combination vs paclitaxel: *p <* 0.001; combination vs AZD8186: *p <* 0.0145).

To test the *in vivo* efficacy of AZD8186 in combination with paclitaxel, MDA-MB-436 and MDA-MB-468 cells were implanted subcutaneously into the mammary fat pad of athymic nude mice. Mice bearing xenografts were treated with either vehicle, AZD8186 (50 mg/kg daily), paclitaxel (10 mg/kg, weekly) or AZD8186 + paclitaxel. Dynamic tumor volume measurement showed that compared to the vehicle control groups where xenograft tumors progressively grew within the experimental span, AZD8186 did not cause significant tumor growth inhibition as a single agent in both cell line models (Figure 5A, 5B). However, AZD8186 showed synergistic combination efficacy with paclitaxel, with CIs of 0.473 and 0.788, respectively, in each xenograft model. In MDA-MB-436 xenograft, AZD8186 in combination with paclitaxel was significantly more effective than vehicle or either single agent AZD8186 or paclitaxel (combination vs vehicle: *p =* 0.0049; combination vs AZD8186: *p <* 0.001; combination vs paclitaxel: *p =* 0.0104; Figure 5A). We noticed that, despite the best response to the combination of AZD8186 with paclitaxel, tumor growth was still progressive in this TNBC model. In MDA-MB-468 model, combinatory treatment with AZD8186 and paclitaxel was significantly more effective than vehicle or either single agent AZD8186 or paclitaxel (combination vs vehicle: *p <* 0.001; combination vs AZD8186: *p =* 0.0145; combination vs paclitaxel: *p =* 0.001). Further, this combination achieved a substantial stabilization of the growth rate compared to vehicle control with a T/C ratio 0.33 (tumor volume of Treatment group / tumor volume of Control group × % at the end time point).

### AZD8186 in combination with anti-PD1 inhibition has enhanced antitumor efficacy *in vivo*

**Figure 6:**
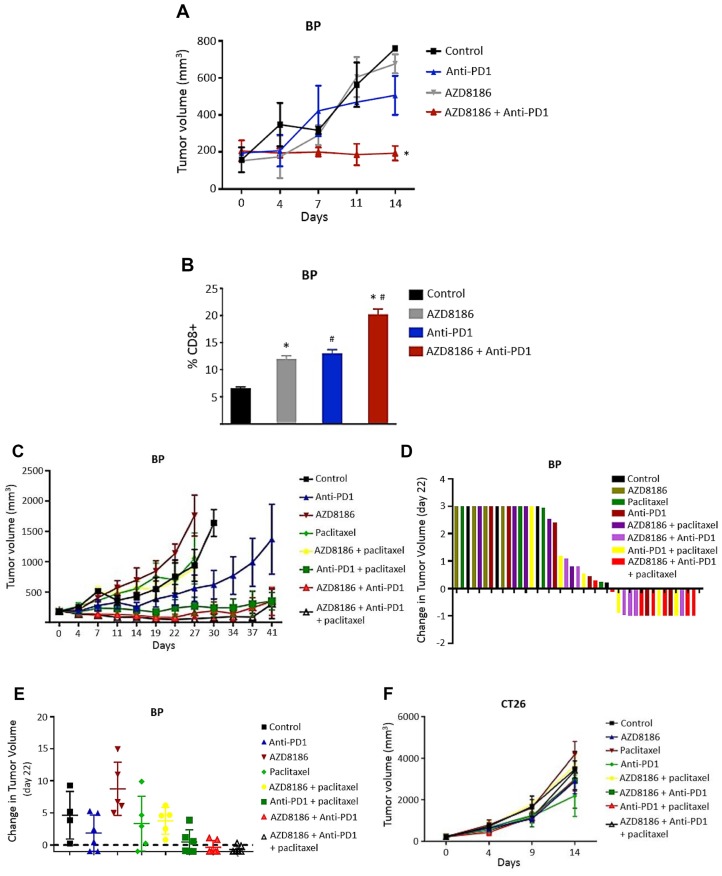
Effects of AZD8186 in combination with anti-PD1 on syngeneic models. (**A**) Xenograft of murine melanoma BP cells. BP cells were subcutaneously implanted into BALB/c mice. Mice bearing BP xenografts were treated with vehicle, AZD8186 at 50 mg/kg daily, anti-PD1 at 200 μg, twice a week or AZD8186 in combination with anti-PD1 for 14 days. Tumor volume was measured at the indicated time points. Mean ± SEM values of tumor volume were obtained from 5 mice in each treatment group from single experiment. The tumor volumes at the conclusion of experiment were analyzed by two-way analysis of variance to determine statistical significance. ^*^ *p* values (combination vs control: *p <* 0.001; combination vs AZD8186: *p =* 0.002; combination vs anti-PD1: *p =* 0.003). (**B**) Mass cytometry analysis. Tumors were harvested from the same mice above at the end point. Tumors were digested and stained with mass cytometry staining antibody panel including CD8 antibody. Samples were then analyzed using a CyTOF2 or Helios mass cytometer. Values are presented as mean ± SEM of percentage of CD8+ TILs of tumors. Data were analyzed by multiple t-tests to determine statistical significance. ^*^ *p =* 0.02 (AZD8186 vs control); ^#^ *p =* 0.016 (anti-PD1 vs control); ^*#^ *p* values for combination (combination vs control: *p =* 0.001; combination vs AZD8186: *p =* 0.01; combination vs anti-PD1: *p =* 0.014). (**C**, **F**) Xenografts of BP cells (C) and CT26 cells (F). BALB/c mice bearing xenografts were treated with vehicle, AZD8186 at 50 mg/kg daily, anti-PD1 at 200 μg, twice a week, paclitaxel at 10 mg/kg, once a week, and double or triple combinations. Values are presented as mean ± SEM of tumor volume. The methods for tumor measurement and statistical analysis were the same as (A). *p* values for (C) are: AZD8186 vs control: *p =* 0.037; anti-PD1 vs control: *p =* 0.001; AZD8186+anti-PD1 combination vs AZD8186: *p <* 0.0001; AZD8186+anti-PD1 combination vs anti-PD1: p 0.0011; AZD8186+anti-PD1 combination vs control: *p =* 0.0002. (**D**, **E**) Waterfall of tumor volume changes in all the animals (D) and mean changes of tumor volume (E) for (C).

*In vivo* antitumor efficacy of AZD8186 in combination with RPM1-14 (anti-mouse PD1 monoclonal antibody; anti-PD1) was evaluated in a syngeneic PTEN-deficient mouse cell model, BP (murine melanoma). Cells were implanted subcutaneously into BALB/c mice. When tumors reached at least 100 mm^3^, mice were treated with either vehicle, AZD8186 (50 mg/kg daily), anti-PD1 (200 μg, twice a week) or AZD8186 + anti-PD1. We found that at day 14 of treatment, while AZD8186 and anti-PD1 alone moderately reduced tumor volume with T/C ratio 84.4% and 74.7%, respectively, combination treatment demonstrated significantly greater antitumor efficacy with T/C ratio 26.2%, than vehicle (*p <* 0.001), or AZD8186 (*p =* 0.002), or anti-PD1 (*p =* 0.003) 01) (Figure 6A). Bliss analysis showed that combination of AZD8186 with anti-PD1 created a powerful synergistic tumor inhibitory effect with CI of 0.499. Next, we sought to determine the potential mechanism for such a synergistic combination. PTEN loss is known to contribute to immune resistance by decreasing T cell tumor infiltration [17]. Thus, we examined the effects of drug combination on CD8+ tumor infiltration lymphocytes (TILs). The tumor tissues harvested from the same treated mice above were used for mass cytometry analysis. Results showed that when single drug treatment moderately increased the percentage of CD8+ TILs in the tumors (AZD8186 vs control: *p =* 0.02; anti-PD1 vs control: *p =* 0.016), combination treatment was associated with significantly higher percentage of CD8+ cells than vehicle control (*p =* 0.001), or AZD8186 alone (*p =* 0.01), or anti-PD1 alone (*p =* 0.014) (Figure 6B).

In a separate mice experiment, we compared the combination efficacy between PTEN-loss and PTEN-WT models and also tested if chemotherapy enhances immune efficacy in the presence of AZD8186. Mice bearing either PTEN-deficient BP xenografts or PTEN-WT colon carcinoma CT26 xenografts were treated with vehicle, AZD8186 (50 mg/kg daily), anti-PD1 (200 μg, twice a week), paclitaxel (10 mg/kg, once a week), or various combinations between these agents as indicated in Figure 6C. Again, anti-PD1 alone moderately decreased tumor growth with T/C ratio of 61.1% (*p =* 0.001). However, when anti-PD1 was combined with AZD8186, it demonstrated significantly greater antitumor efficacy than single AZD8186 (*p <* 0.0001), or anti-PD1 (*p =* 0.0011), or vehicle control (*p =* 0.0002), with a T/C ratio 9.2% (Figure 6C). These lead to a strong combinatory synergy with CI of 0.551. Consistently, these results further confirmed the synergistic efficacy on tumor growth inhibition by combination of AZD8186 with anti-PD1 that we found in Figure 6A. We also found that combination of anti-PD1 with paclitaxel, or with paclitaxel and AZD8186, exhibited substantially enhanced antitumor efficacy compared to single agent treatments. Among them, tumor volume in three-drug combination group was the smallest compared to all other groups (Figure 6C). All three anti-PD1 combination groups achieved disease stabilization. Furthermore, a tumor regression was observed in anti-PD1 + AZD8186 and three-drug combination groups. Compared to the initial tumor volume, tumor volume was reduced by about 50% in these two groups across this treatment period (Figure 6C). These synergistic antitumor efficacy of AZD8186 with paclitaxel and with anti-PD1 was also presented by a waterfall plot of tumor volume changes in all the animals (Figure 6D) and by mean changes of tumor volume (Figure 6E).

On the other hand, no significantly enhanced antitumor efficacy was appreciated in all the combination cohorts in the PTEN-wild-type CT26 model (Figure 6F). Interestingly in CT26, all treatments also resulted in an increase in CD8+ TILs compared to the controls; however, there was not a statistically significant difference between the groups (data not shown).

## DISCUSSION

TNBC is defined as tumors lacking hormone receptors (estrogen and progesterone) and HER2 expression and represents approximately 15–20% of all breast cancer patients and is associated with a poor prognosis [23]. Patients with TNBC who have residual disease following neoadjuvant chemotherapy are at high risk of relapse and have few options upon recurrence [24, 25]. Therefore, there is a great need for superior therapy options for TNBC. PTEN loss is common in breast cancer [8, 26]. Haddadi *et al* reported that up to 30% of breast cancer patients have PTEN loss [12]. Deregulatory activation of PI3K-Akt signaling from loss of PTEN negative regulation is known as a significant contributor to pathogenesis of cancer. Previous work has shown that PTEN-deficient tumors are dependent on PI3Kβ activity [11]. Therefore, we explored the possibility of targeting PI3Kβ with AZD8186 for therapeutics of PTEN-deficient TNBC.

Our *in vitro* evaluation of AZD8186 demonstrated selective sensitivity in PTEN-deficient TNBC cell lines, as evidenced by selective inhibition of proliferation and PI3K signaling, and stimulation of apoptosis in these cells. However, *in vivo* evaluation demonstrated limited single agent efficacy of AZD8186 on tumor growth inhibition at a clinically achievable dose in PTEN-deficient cell line xenografts noted to be sensitive *in vitro*. No tumor regression was observed in either TNBC model.

There is little information about factors that limit single agent clinical efficacy of AZD8186. Although PI3Kβ is the primary PI3K isoform involved in many cases of tumorigenesis that are driven by PTEN loss, studies have shown that depending on the tissue type and pathology, both PI3Kα and PI3Kβ may be involved [27–29]. Previous studies have also identified a feedback loop between PI3Kα and PI3Kβ [1, 30]. It was found that reactivation of phospho-AKT following AZD8186 treatment could result from a feedback reactivation of other PI3K isoforms, particularly PI3Kα. Other evidence have indicated that prolonged treatment of PTEN-deficient tumor cells with PI3Kβ-selective inhibitors can shift isoform dependency from PI3Kβ to PI3Kα. In addition, variations in multiple cellular pathways that are associated with pharmacological mechanisms of AZD8186, such as the cellular carbon metabolism pathway involving pyruvate dehydrogenase kinase 4 (PDHK4), and the DNA repair pathway involving replication protein A 32 kDa subunit (RPA32) and phosphorylated histone protein H2AX (γH2AX) [15], may also contribute to the limited efficacy of AZD8186.

Given the limited efficacy, we sought to seek effective combination approaches in AZD8186 therapeutics in TNBC tumors. First, we explored combination of AZD8186 with chemotherapeutic agents. *In vitro* combination assays in the TNBC cell lines revealed that combinations of AZD8186 with chemotherapy agents have enhanced inhibition efficacy on cell proliferation and cell apoptosis. These data suggest that chemotherapeutic agents could be effective partners for AZD8186 in TNBC therapy. Moreover, our *in vivo* TNBC tumor models demonstrated a strong therapeutic synergy of combination between AZD8186 and paclitaxel. These finding are consistent with the results of a previous study where AZD8186 synergized with microtubule inhibitor docetaxel on tumor growth inhibition in xenograft of a PTEN-deficient cell line, HCC-70 breast cancer cells [12]. However, in MDA-MB-436 model, progressive disease was still observed. In addition, no increased antitumor efficacy was observed in patient-derived xenografts (data not shown). The potential reasons for variation in combination efficacy may include tissue type, coexisting genetic events and microenvironment cues that fuel cancer cells. Therefore, other combination therapies were evaluated.

In addition to PI3Kβ inhibition, AZD8186 also has activity against PI3Kδ. Although PI3Kδ does not function as a pivotal driver in PTEN-deficient tumors [11], inhibition of PI3Kβ in the tumor cells and PI3Kδ in the immune suppressive myeloid cells with AZD8186 provides a complementary approach to restoring antitumor immunity and enhancing effector T-cell function [31]. Previous reports described increased PD-L1 expression induced by PTEN loss in TNBC, suggesting PD-1 inhibitor therapy as an interesting treatment for PTEN-null tumors [16]. However, other studies found that loss of PTEN is associated resistance to T cell-mediated immunotherapy with anti-PD1 [17, 32]. This provides a strong rationale to explore the combination efficacy between AZD8186 and anti-PD1 on PTEN-deficient tumors.

Our *in vivo* data demonstrated that the combination produced a strong synergistic antitumor efficacy compared to AZD8186 or anti-PD1 alone, and resulted in tumor regression in PTEN-deficient BP model, while no increased antitumor efficacy of the combination therapy was appreciated in PTEN-WT CT26 model. Next, we sought to elucidate the mechanisms underlying such a combination efficacy. Previously, PTEN loss was found to be correlated with decreased T cell tumor infiltration, and loss of PTEN inhibits T cell-mediated tumor killing in preclinical models [17]. Therefore, we evaluated the status of CD8+ lymphocyte infiltration. Mass cytometry measurement of PTEN-deficient BP tumors revealed an increased CD8+ lymphocyte infiltration of the tumors at the conclusion of the therapy by combination of AZD8186 and anti-PD1 compared to the control and single agent AZD8186. Interestingly in the PTEN-WT CT26, all single agent treatment groups resulted in an increase in CD8+ TILs compared to the control cohort; however, there was not a statistically significant difference between the single agent groups (data not shown). These results are consistent with our hypothesis that combination between PI3Kβ inhibitor AZD8186 and immune checkpoint inhibitor anti-PD1 has a synergistic therapeutic efficacy in PTEN-deficient tumors. Further work is needed to get additional mechanistic insights and to determine whether this combination is also effective in other tumor types with PTEN loss of function.

In conclusion, these results provide preclinical evidence of antitumor efficacy of AZD8186 in PTEN-deficient solid tumors. AZD8186 has single agent efficacy in PTEN-deficient TNBC cell lines *in vitro*, with modest single agent efficacy *in vivo*. Furthermore, AZD8186 enhanced the antitumor efficacy of paclitaxel but stable and progressive disease were noted with this combination in immunosuppressed models. In immunocompetent models, AZD8186 in combination with anti-PD1 resulted in tumor regression in PTEN-deficient BP tumor. We realize that while there appears to be an association of AZD8186 sensitivity to PTEN loss, a cause effect relationship can only be speculated on. In summary, although further insights are needed into the mechanisms of activity of these combinations, the combination of AZD8186 with taxanes and with anti-PD1 agents hold promise for the treatment of PTEN-deficient solid tumors.

## MATERIALS AND METHODS

### Cell lines, drugs, and antibodies

Breast cancer cell lines BT-20, BT-549, HCC-1806, HCC-1937, HCC-38, MDA-MB-231, MDA-MB-436, and MDA-MB-468 were obtained from American Type Culture Collection (ATCC). Breast cancer cell line MFM-223 was purchased from Sigma. Sum-159 breast cancer cell line generation was previously described [33]. All these cells were cultured in Dulbecco’s modified Eagle’s medium/F-12 supplemented with 10% fetal bovine serum at 37° C and in a humidified incubator containing 5% CO_2_.

Paclitaxel and carboplatin were obtained from Selleck Chemicals. Eribulin was acquired from D Anderson Cancer Center’s pharmacy (Houston, TX, USA). For *in vitro* studies AZD8186 and AZD5363 were obtained from Selleck Chemicals. For *in vivo* studies AZD8186 was obtained from AstraZeneca as a generous gift. Anti-mouse PD1 monoclonal antibody (clone: RPM1-14) was purchased from BioXcell. For *in vitro* studies, all drugs were dissolved in DMSO.

Immunoblotting antibodies purchased from Cell Signaling Technology (CST) include anti-phospho-AKT (S473) (#4060), anti-AKT (#9272), anti-phospho-S6K (T389) (#9234), anti-S6K (#9202), anti-phospho-PRAS40(T246) (#2997), and PRAS40 (#2610). Anti-β-actin antibody (#A5441) was purchased from Sigma. Secondary antibodies Goat-anti-Rabbit-Alexa Fluor-680 (#A21076) and Goat-anti-Mouse-Dyligh-800 (#610145-121) were purchased from Life Tech and Rockland Immunochemicals, respectively.

### Cell growth assay

Cells were seeded in 96-well plates at densities of 5000 to 10000 cells per well depending on growth characteristics of each cell line. After adhering overnight, titrating concentrations of the designated drug were added to the wells in triplicates and incubated at 37° C for 72 hours. Anti-proliferative activity was evaluated by sulforhodamine B (SRB) assay. The half maximal inhibitory concentration (IC_50_) and combination index (CI) were determined from dose-response curves generated using GraphPad Prism v6.05 software. All experiments were repeated at least three times.

### Colony formation assay

Cells were plated at a density of 2 × 10^3^ cells in 60 mm plates in triplicate for each treatment group. Cells were treated the next day with the indicated concentrations of AZD8186, vehicle control (DMSO) for two weeks. The culture medium was changed every seven days. The colonies were then fixed in 10% formalin and stained with 0.05% crystal violet in 25% methanol. Percent surface area was measured using NIH ImageJ v.1.48 software.

### Western blot analysis

Cells were washed with cold PBS and lysed in Laemmli buffer. The protein was quantified using Pierce BCA protein assay Kit (ThermoFisher) before loading to the gel. After SDS-PAGE, the protein was transferred to a 0.2 μm nitrocellulose membrane (Bio-Rad Laboratories). Membranes were blocked with 0.1% casein in TBS. Immunoblotting was performed with the following antibodies: PTEN, PI3Kβ, AKT, phospho-AKT, S6K, phospho-S6K, PRAS40, phospho-PRAS40, and β-actin. The immunoblots were visualized using the Odyssey IR imaging system (Li-Cor Biosciences). Representative blots of at least 2 independent experiments are shown.

### Cell cycle and apoptosis assays

Cells were plated and allowed to attach to the petri dish overnight. The following day, cells were treated with DMSO or AZD8186 in triplicates. After 72 hours, floating and attached cells were collected. DNA content was determined in flow cytometry using propidium iodide (Roche) following manufacturer’s protocol. Apoptosis was identified by using the Annexin V apoptosis kit (Roche) according to the manufacturer’s protocol. Samples were analyzed by flow cytometry at The Flow Cytometry and Cellular Imaging Core Facility at MD Anderson Cancer Center.

### *In vitro* combination assay

Four TNBC cell lines MDA-MB-436, MDA-MB-468, Sum-159 and MFM-223 were treated with each drug at six concentrations (5× dilution), starting the highest concentration at 25 μM for AZD8186, 100 nM for paclitaxel, 5 nM for eribulin, and 100 μM for carboplatin, and with drug combinations at each six concentrations, for 72 hours. Sulforhodamine B staining was performed to measure cell viability rate. CI was calculated using Chou and Talalay’s combination model based on IC50s of single drug treatment and combination treatment. CI values: <0.8: synergism; 0.8 – 1.2: addition; and >1.2: antagonism.

### *In vivo* treatment

For *in vivo* experiments, AZD8186 was prepared in 10% DMSO/60% tri-ethylene glycol (TEG)/30% water, and paclitaxel was diluted to appropriate volume in PBS prior to administering to mice. RPM1-14 (Anti-mouse PD1 monoclonal antibody; Anti-PD1) was diluted in appropriate volume of InVivoPure pH7.0 dilution buffer. Female athymic nude mice were used for inoculation of human breast cancer cell lines MDA-MB-436 or MDA-MB-468. BALB/c mice were used for murine cell lines BP (BRAF and PTEN mutant melanoma) and CT26 (PTEN wild-type colon carcinoma). 5 × 10^6^ cells were subcutaneously inoculated into the mammary fat pad. Drug treatments were started once tumors reached at least 100 mm^3^. AZD8186 was orally administrated at 50 mg/kg daily; paclitaxel was intravenously injected at 10 mg/kg, once a week; Anti-PD1 was administrated by intraperitoneal injection at 200 μg, twice a week. Each treatment group has five mice. Tumor volume (TV) was measured at different time points using the formula: TV (mm^3^) = ((width)^2^ × length)/2. T/C ratio at the end time point was calculated using formula: TV of treatment group / TV of control group × %. Combination Index (CI) for tumor inhibition was calculated using Bliss Independence combination model [34–36]. Formula: CI = [(Ea + Eb) – (Ea × Eb)] / Eab. Ea and Eb are tumor growth inhibition rate with drug “a” and “b”; Eab is tumor growth inhibition rate with combination. Tumor growth inhibition rate = 1 − T/C ratio.

### Mass cytometry analysis of murine tumors

Tumors were dissected, manually dissociated, and digested enzymatically with Liberase TL (Roche) and DNase I (Roche) in RPMI-1640 for 30 min at 37° C. Digested tumors were then run through 70 μm filters into RPMI-1640 supplemented with 10% FBS, sodium pyruvate, β-mercaptoethanol, and penicillin and streptomycin. Single cell suspensions were then purified on a Histopaque-1119 (Sigma-Aldrich) gradient centrifuged at 2000 rpm for 20 min at room temperature [37]. Cells were then washed twice with FACS buffer (fresh DNAase-containing Iscove’s medium with 5% heat-inactivated FCS, Hepes and Glutamax) and total cell concentration determined. 3 × 10^6^ cells per tumor were then incubated with fluorescence-conjugated anti-mouse CD16/CD32 antibody (2.4G2) in PBS with 2% of each bovine, murine, rat, hamster, and rabbit serum at 4° C for 10 min [37]. Cells were stained for surface antibodies with an antibody cocktail at 4° C for 30 min in a 50 μl volume. Cells were incubated with 2.5 μM 194Pt monoisotopic cisplatin (Fluidigm) at 4° C for 1 min. Cells were washed twice with FACS buffer. Cells were then fixed and permeabilized using the Foxp3 fix and permeabilization kit according to manufacturer’s protocol (eBioscience). Cells were subsequently stained with an intracellular stain antibody cocktail for 30 min at room temperature. Cells were then washed twice with Foxp3 permeabilization buffer, twice with FACS buffer, and incubated overnight in 1.6% PFA PBS with 100 nM Iridium nucleic acid intercalator (Fluidigm). Cells were then washed twice with 0.5% BSA PBS, filtered, and washed twice with 0.1% BSA water prior to analysis. Samples were then analyzed using a CyTOF2 or Helios mass cytometer using the Helios 6.5.358 acquisition software (Fluidigm). Anti-mouse CD8a (clone 53-6.7) was used for CD8 analysis of BP and CT26 TILs in checkpoint blockade experiments.

### Statistical analysis

For *in vitro* studies, Student t-test was performed to compare between two groups, while 1-way ANOVA followed by Tukey multiple comparison tests was performed to compare multiple groups. Association between PTEN protein status and AZD8186 sensitivity was tested with Fisher’s exact test. For the *in vivo* study, two-way ANOVA tests followed by Tukey for multiplicities. Data was presented as mean ± SEM.

## SUPPLEMENTARY MATERIALS



## Abbreviations

PI3K: Phosphatidylinositol 3-kinase
PTEN: Phosphatase and tensin homolog
TNBC: Triple-negative breast cancer
PD-L1: Programmed death-ligand 1
shRNA: Small hairpin RNA
DMSO: Dimethyl sulfoxide
SRB: Sulforhodamine B
PAGE: Polyacrylamide gel electrophoresis
CI: Combination index
PDX: Patient-derived xenograft
