# Supplementary material for: Targeting PI3Kβ alone and in combination with chemotherapy or immunotherapy in tumors with PTEN loss
Source: Oncotarget. 2020 Mar 17;11(11):969–81. doi: 10.18632/oncotarget.27503 (PMC7082117; doi:10.18632/oncotarget.27503)
Supplement: Supplementary file 1 [file oncotarget-11-969-s001.pdf]

Targeting PI3Kβ alone and in combination with chemotherapy or immunotherapy in tumors with PTEN loss

SUPPLEMENTARY MATERIALS

Supplementary Table 1: AZD8186 IC50 in 10 TNBC cell lines

Cell line	AZD8186 IC50 (nM)	
	Mean	SD
BT-549	31	27
MDA-MB-468	358	328
MDA-MB-436	899	252
HCC-1937	8993	399
MDA-MB-231	6985	519
BT-20	2816	124
HCC-1806	3645	209
Sum-159	4385	421
MFM-223	14311	1076
HCC-38	5238	342

Supplementary Table 2: AZD8186 combination index (*in vitro*)

Cell line	Test Date	Combination index of AZD8186 with		
		**Paclitaxel**	**Eribulin**	**Carboplatin**
**MDAMB436**	30-May	0.33	0.56	
	3-Jun			
	7-Jun	0.19	0.35	0.20
	9-Dec	0.58	0.64	0.60
	Average	**0.37**	**0.52**	**0.40**
	STDEV	**0.20**	**0.15**	**0.28**
**MDAMB468**	30-May	0.18	0.32	
	3-Jun	0.31	0.39	
	7-Jun	0.62	0.67	1.05
	9-Dec	0.14	0.20	0.53
	Average	**0.31**	**0.40**	**0.79**
	STDEV	**0.22**	**0.20**	**0.37**
**Sum159**	30-May	0.53	1.24	
	3-Jun	0.39	0.32	
	7-Jun	0.48	0.34	0.67
	9-Dec	0.43	0.50	0.33
	Average	**0.46**	**0.60**	**0.50**
	STDEV	**0.06**	**0.43**	**0.24**
**MFM223**	30-May	0.63	0.79	
	3-Jun	0.36	0.52	
	7-Jun	1.43	1.57	1.07
	9-Dec	0.26	0.54	0.55
	Average	**0.67**	**0.86**	**0.81**
	STDEV	**0.53**	**0.49**	**0.37**
